# Case Report: Navigating the bleeding-thrombosis paradox: regional nafamostat anticoagulation in a post-intracerebral hemorrhage patient on VV-ECMO

**DOI:** 10.3389/fmed.2026.1840332

**Published:** 2026-07-15

**Authors:** Shujia Zhang, Zhenyin Zhang, Jin Yang, Jiangqiong Peng, Yi Yang, Longxiang Su, Jing Jiang

**Affiliations:** 1 Department of Critical Care Medicine, Chongqing Academy of Medical Sciences, Chongqing General Hospital, Chongqing University, Chongqing, China; 2 Department of Critical Care Medicine, State Key Laboratory of Complex Severe and Rare Diseases, Peking Union Medical College Hospital, Chinese Academy of Medical Sciences and Peking Union Medical College, Beijing, China

**Keywords:** acute respiratory distress syndrome, anticoagulation, extracorporeal membrane oxygenation, intracerebral hemorrhage, nafamostat

## Abstract

Extracorporeal membrane oxygenation (ECMO) in patients following intracerebral hemorrhage (ICH) poses a critical dilemma between circuit thrombosis and catastrophic rebleeding. While nafamostat mesylate (NM) has been increasingly explored for ECMO anticoagulation in patients with ICH, it is conventionally administered as a systemic anticoagulant with high activated partial thromboplastin time (aPTT) targets, which still carries significant bleeding risks. Moreover, whether NM can achieve effective regional anticoagulation in venovenous (VV) ECMO remains highly controversial. Here, we present a post-ICH patient with severe ARDS who required VV-ECMO. Due to suspected rebleeding, anticoagulation was transitioned from heparin to an anticoagulant-free strategy, which subsequently led to significant oxygenator thrombosis. To navigate this crisis, NM was initiated with an ultra-low systemic target, but it initially failed to establish a regional anticoagulant effect when infused at the conventional pre-membrane, post-pump site. Crucially, relocating the NM infusion to the pre-pump position successfully established a significant circuit-to-systemic aPTT gradient. This technical optimization enabled effective circuit anticoagulation while minimizing systemic bleeding risk, allowing for successful ECMO weaning without bleeding complications. This dynamic intra-patient observation highlights the potential impact of infusion site on coagulation outcomes. We hypothesize that harnessing the centrifugal pump’s high-shear turbulence for homogenous drug mixing might serve as a crucial prerequisite for achieving regional anticoagulation in VV-ECMO. These findings bridge the gap between theoretical pharmacology and practical circuit engineering, offering a practical preliminary reference for the future anticoagulant management of similar high-bleeding-risk patients on ECMO.

## Introduction

Spontaneous intracerebral hemorrhage (ICH) is frequently complicated by acute respiratory distress syndrome (ARDS), contributing to high mortality and elevated treatment costs (1). Extracorporeal membrane oxygenation (ECMO) serves as a salvage therapy for severe ARDS refractory to conventional mechanical ventilation. However, the systemic anticoagulation required for ECMO poses a critical clinical dilemma, as it may increase the risk of ICH recurrence and mortality (2). Currently, no standardized anticoagulation strategy exists for post-ICH patients on ECMO support. Some centers have attempted low-dose heparin or anticoagulant-free protocols, but supporting evidence remains limited (3).

Nafamostat mesylate (NM), a broad-spectrum serine protease inhibitor with an ultra-short half-life (5–8 min), has emerged as an alternative anticoagulant for high-bleeding-risk patients requiring ECMO support or blood purification (4). However, in most current clinical practices, including a recent cohort of post-ICH patients (5), NM is conventionally administered as a systemic anticoagulant with relatively high activated partial thromboplastin time (aPTT) targets. Such systemic exposure and high targets still carry a non-negligible bleeding risk (6). Theoretically, NM’s pharmacokinetic profile makes it an ideal candidate for regional anticoagulation (7), representing potentially the optimal solution to this bleeding-thrombosis paradox. However, whether effective regional anticoagulation can be reliably achieved in venovenous (VV) ECMO remains highly controversial (8, 9).

Herein, we present a high-bleeding-risk post-ICH patient with severe ARDS successfully managed with VV-ECMO using NM anticoagulation. Initially, we also observed a paradoxical failure to achieve regional anticoagulation when the NM infusion was administered at the conventional post-pump site with a low anticoagulation target. Therefore, we strategically relocated the infusion to the pre-pump position to harness the centrifugal pump’s high-shear turbulence for homogenous drug mixing. This crucial adjustment successfully established a strict circuit-to-systemic aPTT gradient, enabling effective control of circuit thrombosis while minimizing systemic bleeding risks. This unique intra-patient observation suggests that regional anticoagulation may be achievable in VV-ECMO, offering potential mechanistic insights into infusion site and providing a practical reference for the future management of similar extreme-bleeding-risk patients.

## Case

A 41-year-old Chinese male with a history of hypertension, diabetes, and chronic kidney disease underwent emergency endoscopic hematoma evacuation for a right basal ganglia hemorrhage (Figure 1A). Postoperatively (Figure 1B), the patient regained consciousness, but left-sided hemiparesis persisted. On day 4, he developed a pulmonary infection and respiratory failure, prompting antimicrobial therapy and tracheostomy. On day 18, due to progressive pneumonia and severe ARDS, he was transferred to the intensive care unit (ICU). Despite maximal medical management including lung-protective ventilation, recruitment maneuvers, positive end-expiratory pressure titration, and prone positioning, his PaO₂/FiO₂ ratio remained at 49 mmHg.

**Figure 1:**
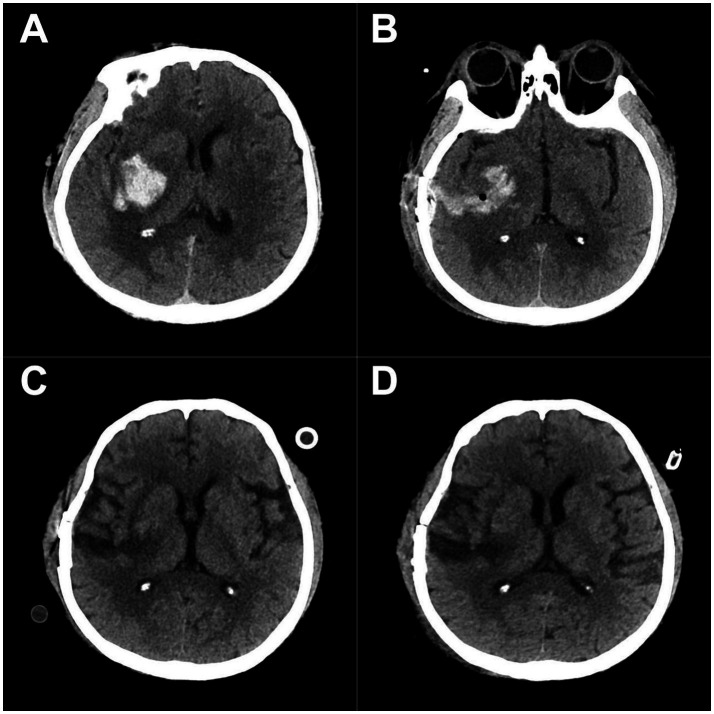
Serial cranial computed tomography (CT) scans illustrating the radiological evolution of the intracerebral hemorrhage throughout the clinical course. **(A)** Initial CT scan demonstrating a right basal ganglia hemorrhage. **(B)** Postoperative CT scan obtained following emergency endoscopic hematoma evacuation. **(C)** Emergency CT scan performed on the morning of day 24 after the episode of acute bilateral pupillary dilation, showing no evidence of recurrent hemorrhage or cerebral herniation. **(D)** Follow-up CT scan obtained on day 30 after ECMO weaning, demonstrating continued hematoma stability. Low-resolution figures are embedded here for review purposes; high-resolution original files have been uploaded separately as required.

Consequently, on day 21, as chest radiography revealed diffuse bilateral consolidation (Figure 2A), venovenous ECMO (VV-ECMO) was initiated combined with continuous renal replacement therapy (CRRT) for progressive anuria. Prior to ECMO cannulation, a heparin bolus of 10 U/kg (87 kg body weight) was administered, followed by a continuous infusion at 500 U/h. Given the recent ICH, we targeted an ultra-low aPTT of 35–45. In the early hours of day 24, the patient developed acute bilateral pupillary dilation (5 mm, non-reactive). Detailed neurological examination was precluded by continuous sedation, analgesia, and muscle relaxation. An urgent neurological consultation suspected recurrent ICH with herniation. However, an emergent CT scan was deferred due to the high risk of transporting an ECMO-dependent patient overnight. Accordingly, heparin was discontinued empirically, and mannitol was administered for suspected intracranial hypertension. A cranial CT performed later that morning revealed no new cerebral hemorrhage or herniation (Figure 1C), while the patient’s pupil size fluctuated between 2 and 6 mm with occasional light reflexes. Given the extreme risk of catastrophic ICH expansion, a no-anticoagulation strategy was maintained following a multidisciplinary discussion that prioritized neurological preservation over circuit durability. However, increased thrombosis was noted in the oxygenator subsequently, accompanied by rising D-dimer and declining platelet count (Figure 3A).

**Figure 2:**
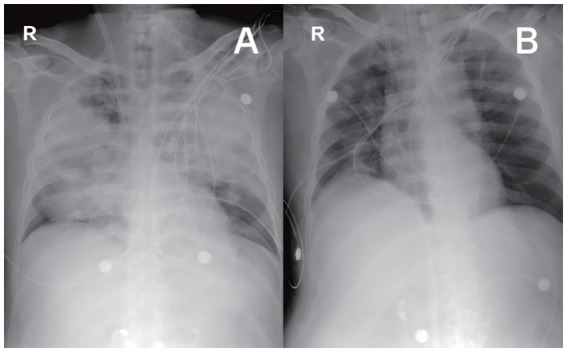
Serial chest radiographs demonstrating lung recovery during ECMO support. **(A)** Chest X-ray on Day 21 (ECMO initiation) showing diffuse bilateral consolidation. **(B)** Chest X-ray on Day 29 (ECMO weaning) revealing significant resolution of pulmonary opacities and improved aeration. ECMO, extracorporeal membrane oxygenation.

**Figure 3:**
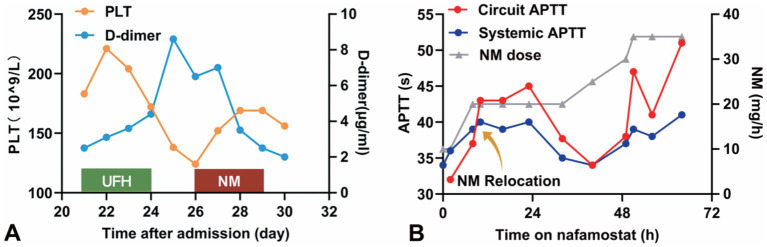
Laboratory trends and NM dosage titration during the clinical course. **(A)** Longitudinal trends of platelet count and D-dimer levels. Transitions from UFH to anticoagulation withdrawal, and subsequently to NM anticoagulation are indicated by color bars. **(B)** NM dosage titration with corresponding circuit and systemic APTT measurements. Relocation of the infusion site (orange arrow) established a significant circuit-systemic APTT gradient (*p* = 0.040). PLT, platelet; UFH, unfractionated heparin; NM, nafamostat mesylate; APTT, activated partial thromboplastin time.

To navigate this bleeding-thrombosis paradox, NM anticoagulation was started on day 26 at 10 mg/h. Following the conventional safety practice in CRRT to avoid negative-pressure air entrainment, the infusion port was initially placed at a pre-membrane, post-pump site. Concurrently, to accurately reflect the true systemic coagulation status and avoid the recirculation artifact typical in VV-ECMO, systemic aPTT was strictly sampled from the radial artery (via a heparin-free flush), while circuit aPTT was drawn from a post-membrane port. Under this dual-monitoring setup, the NM dosage was titrated in 5–10 mg/h increments to maintain an ultra-low systemic aPTT target of 35–45 s, guided by aPTT measurements every 4–6 h. Paradoxically, the initial two aPTT pairs (systemic/circuit: 36/32 s and 39/37 s) failed to show the expected gradient, with systemic aPTT even slightly exceeding circuit aPTT (Figure 3B). Suspecting inadequate drug homogenization due to the proximity of infusion site to the membrane under high ECMO flow rates, we strategically relocated the NM infusion to a pre-membrane and pre-pump position (Figure 4) to harness the centrifugal pump’s high-shear turbulence for homogenous drug mixing. Following this adjustment, circuit aPTT rose markedly above systemic levels (Figure 3B), successfully establishing a robust regional anticoagulation gradient. This was accompanied by the control of circuit thrombosis with parallel improvement in both D-dimer and platelet counts (Figure 3A). As pulmonary conditions significantly improved (Figure 2B), the patient was successfully weaned from ECMO on day 29 (total run time: 224.5 h), without bleeding or systemic thrombotic complications (Figure 1D). He regained consciousness on day 34 and was discharged to a rehabilitation facility on day 54.

**Figure 4:**
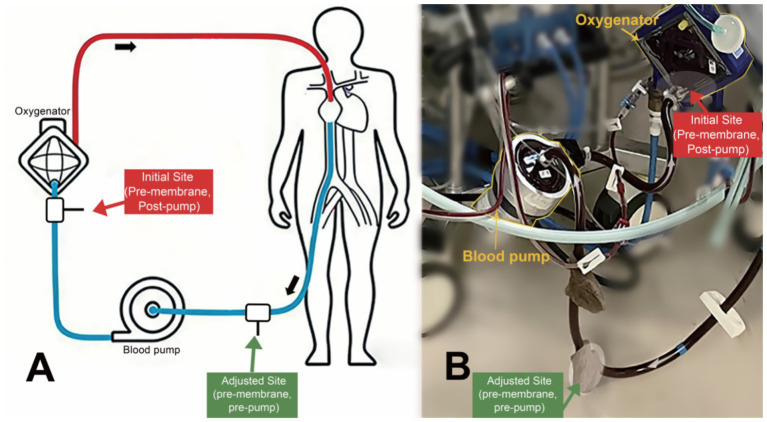
Schematic and photographic representation of NM infusion sites. **(A)** Schematic diagram of the VV-ECMO circuit. **(B)** Bedside photograph of the circuit. NM infusion site was adjusted from a pre-membrane, post-pump position (red) to a pre-membrane, pre-pump position (green). The relocation facilitated superior drug mixing and achieved effective regional anticoagulation. NM, nafamostat mesylate; VV-ECMO, venovenous extracorporeal membrane oxygenation.

## Discussion

Anticoagulation management in post-ICH patients requiring ECMO remains a formidable challenge due to the absence of standardized guidelines. While low-dose heparin or anticoagulation-free strategies have been proposed (3), they are frequently associated with inherent risks of hemorrhage or circuit thrombosis, respectively. Evidence suggests that lower anticoagulation targets may mitigate bleeding risks and improve outcomes (10). Accordingly, given our patient’s bleeding vulnerability (HAS-BLED score of 3), we adopted a tailored, ultra-low systemic aPTT target of 35–45 s. Following the episode of pupillary dilation (retrospectively attributed to rocuronium administration based on the temporal relationship between drug exposure and pupillary changes, together with supporting evidence from the literature (11)), the regimen was adjusted to an anticoagulant-free strategy. Although subsequent cranial CT revealed no recurrent hemorrhage, we maintained this anticoagulant-free strategy after multidisciplinary discussions. This decision prioritized neurological preservation over circuit durability, aligning with current Extracorporeal Life Support Organization (ELSO) guidelines (12) and a recent review (13), which emphasize individualized management and the feasibility of anticoagulant-free approaches, respectively. However, this strategy led to a marked increase in oxygenator thrombosis, underscoring the substantial thrombotic risk without anticoagulation and highlighting the clinical need for a safer alternative to navigate this bleeding-thrombosis paradox.

NM, a broad-spectrum serine protease inhibitor with an ultra-short half-life (5–8 min), is increasingly utilized as an alternative anticoagulant during ECMO or CRRT in patients at high risk of bleeding (4). Recently, a small single-center retrospective study explored its application in ECMO patients complicated with ICH and demonstrated favorable outcomes (5). However, within that cohort, NM was still conventionally administered as a systemic anticoagulant with a relatively high aPTT target of 60–80 s (5). Such systemic exposure and high targets potentially carry a substantial risk of catastrophic rebleeding, a concern corroborated by large retrospective studies reporting even significantly increased bleeding complications with NM compared to heparin (6). Therefore, leveraging NM’s unique pharmacokinetic profile to achieve regional anticoagulation may represent a promising strategy to navigate this bleeding-thrombosis paradox.

Despite its theoretical potential, the feasibility of reliably achieving effective regional anticoagulation with NM during venovenous (VV) ECMO remains a subject of ongoing debate. Park et al. (8) reported a inability to establish a regional aPTT gradient in VV-ECMO, whereas recent Pharmacokinetics/Pharmacodynamics (PK/PD) analyses by Lee et al. (9) demonstrated that the ECMO configuration (VV vs. VA) does not inherently preclude NM’s regional efficacy.

We hypothesize that previous clinical failures in achieving regional anticoagulation during VV-ECMO might partly stem from specific methodological factors, which our case attempted to address. First, as acknowledged by Park et al. (8), sampling systemic blood from the central venous catheter (CVC)—which is anatomically adjacent to the reinfusion cannula in VV-ECMO—can introduce an NM-rich recirculation artifact. In our case, we sought to mitigate this confounder by sampling from the radial artery via a heparin-free flush. Second, *in vitro* studies (14) identified a critical NM concentration threshold (540 ng/mL), beyond which the coagulation time abruptly prolongs. High-dose strategies likely drive both circuit and systemic NM levels above this threshold, inadvertently masking the regional gradient. Our adoption of an ultra-low systemic target (35–45 s) potentially maintained systemic NM concentrations to remain below this safety margin. Finally, our real-time intra-patient observations suggest that extrapolating the routine CRRT habit of post-pump infusion to high-flow ECMO may be insufficient to establish a regional effect, possibly due to inadequate drug homogenization. By strategically relocating the infusion to the pre-pump position, we aimed to harness the centrifugal pump’s high-shear turbulence to thoroughly mix the drug. This crucial adjustment successfully established a distinct regional aPTT gradient, providing a mechanistic rationale that regional anticoagulation may be attainable in VV-ECMO when sampling methodology, pharmacological thresholds, and circuit fluid dynamics are rigorously respected.

Beyond its application for anticoagulation during extracorporeal life support, NM is also indicated for the treatment of acute pancreatitis and disseminated intravascular coagulation (DIC) owing to its inhibitory effects on trypsin and plasmin (3). Clinical evidence suggests that NM may attenuate the severity of acute pancreatitis and improve clinical outcomes (15). In patients with hyperfibrinolytic DIC, particularly those with a high risk of bleeding, NM may have advantages over unfractionated heparin (16). NM has also shown efficacy comparable to gabexate mesilate in the treatment of DIC associated with hematological malignancies (17). Therefore, NM may offer potential advantages in patients with acute pancreatitis or DIC who require blood purification therapy, as it may provide both anticoagulation and adjunctive treatment of the underlying disease (18).

Nevertheless, potential drug interactions and adverse effects should be considered during NM administration. First, concomitant use with other antithrombotic or anticoagulant agents may exert synergistic effects (19, 20), thereby increasing the risk of bleeding and necessitating careful monitoring and dose titration. Second, NM may induce hyperkalemia through reversible inhibition of amiloride-sensitive sodium conductance in the renal cortical collecting ducts (21). Consequently, concurrent administration with other medications that may elevate serum potassium levels, such as potassium-sparing diuretics or angiotensin-converting enzyme inhibitors, requires stringent electrolyte monitoring to prevent potentially fatal arrhythmias. Lastly, caution is warranted when NM is co-administered with certain antibiotics, such as linezolid, as the antioxidant properties of NM may inhibit drug metabolism and delay clearance (22), potentially increasing the risk of adverse effects in critically ill patients.

Several limitations of this study should be considered. First, as a single-case report, the observations are hypothesis-generating and require confirmation in additional patients. Second, the assessment of regional anticoagulation was based solely on aPTT gradients, without direct measurement of nafamostat concentrations in either the systemic circulation or the extracorporeal circuit. Such measurements would have provided more robust pharmacokinetic evidence to support the proposed mechanism. In addition, it remains uncertain whether this pre-pump infusion strategy would produce comparable fluid dynamic effects across different ECMO systems and operating conditions. Further prospective studies incorporating direct nafamostat concentration measurements and PK/PD analyses, are warranted to further evaluate this fluid dynamics-based approach and to determine its applicability in patients at high risk of bleeding.

## Conclusion

In conclusion, this case suggests that regional anticoagulation with NM may be clinically achievable in VV-ECMO, offering a potentially safe and viable strategy for patients with extreme bleeding risks, such as those with recent ICH. Our dynamic intra-patient observations emphasize that achieving this delicate balance extends beyond pharmacology alone; it requires meticulous attention to circuit fluid dynamics, pharmacological thresholds, and rigorous monitoring methodology. These findings provide a valuable practical for managing similar high-risk cohorts, though further prospective studies are warranted to validate this approach.

## Data Availability

The original contributions presented in the study are included in the article/Supplementary material, further inquiries can be directed to the corresponding authors.
