# Supplementary material for: Case Report: Navigating the bleeding-thrombosis paradox: regional nafamostat anticoagulation in a post-intracerebral hemorrhage patient on VV-ECMO
Source: Front Med (Lausanne). 2026 Jul 15;13:1840332. doi: 10.3389/fmed.2026.1840332 (PMC13416251; doi:10.3389/fmed.2026.1840332)
Supplement: Supplementary file 1 [file Presentation_1.pptx]

## Slide 1
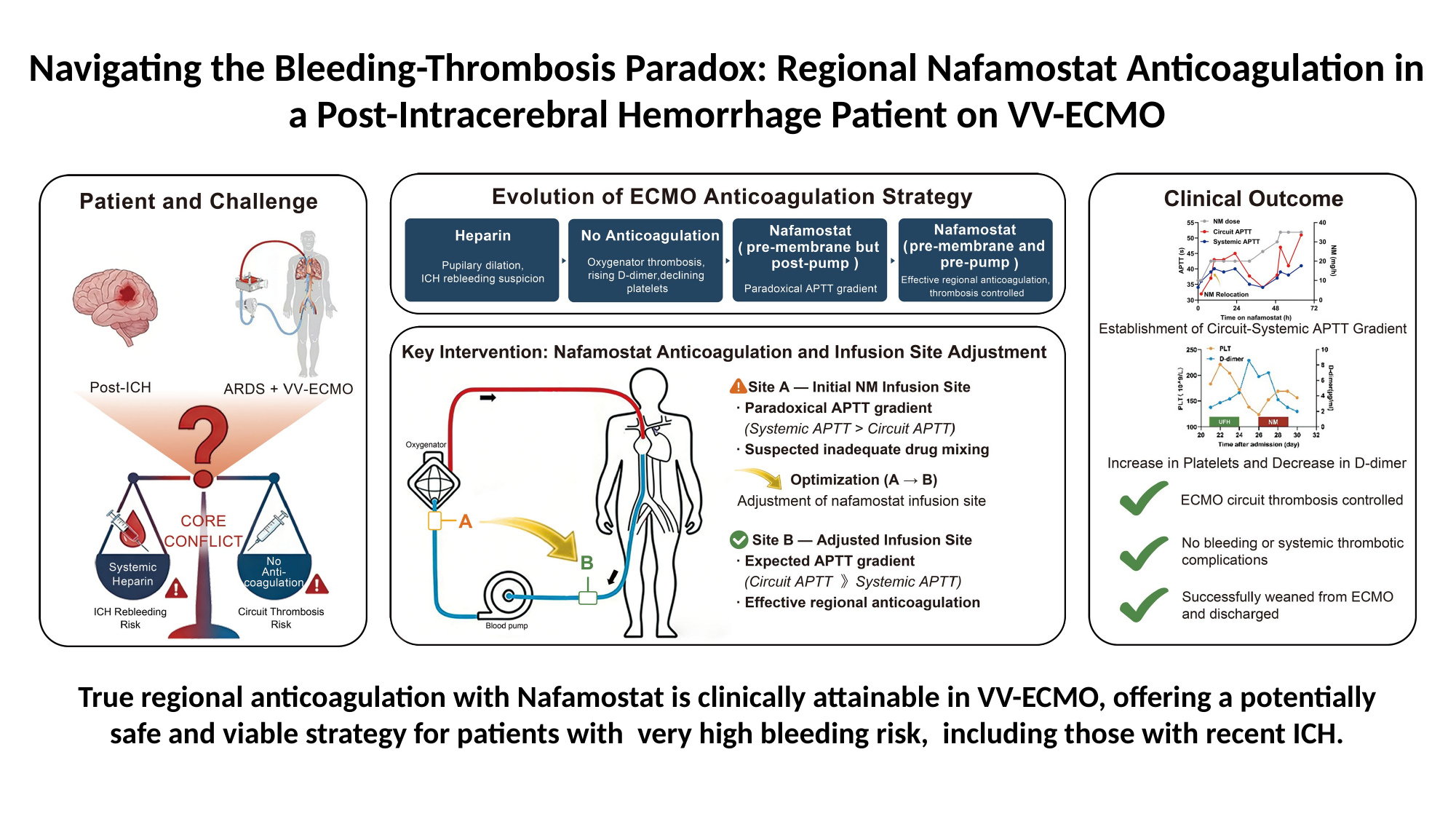

Navigating the Bleeding-Thrombosis Paradox: Regional Nafamostat Anticoagulation in a Post-Intracerebral Hemorrhage Patient on VV-ECMO
True regional anticoagulation with Nafamostat is clinically attainable in VV-ECMO, offering a potentially safe and viable strategy for patients with very high bleeding risk, including those with recent ICH.
